# Feasibility of caregiver-administered anthropometric measurements of children under age 5: evidence from Zambia

**DOI:** 10.1186/s12963-024-00322-4

**Published:** 2024-01-31

**Authors:** Günther Fink, Mpela Chembe, Savanna Henderson, Peter C Rockers, Doug Parkerson

**Affiliations:** 1 Swiss Tropical and Public Health Institute and University of Basel, Kreuzstrasse 2, 4123 Allschwil, Basel, Switzerland; 2 Innovations for Poverty Action Zambia, Lusaka, Zambia; 3 Innovations for Poverty Action, Washington, DC USA; 4 School of Public Health, Boston University, Boston, USA; 5 Innovations for Poverty Action, New Haven, USA

**Keywords:** Anthropometry, Measurement, Self-measurement, Caregiver-administered measurement, Height, Weight, MUAC

## Abstract

**Background:**

Accurate measurement of children’s anthropometry is of central importance for the assessment of nutritional status as well as for the evaluation of nutrition-specific interventions. Social distancing requirements during the recent Covid-19 pandemic made administration of standard assessor-led measurement protocols infeasible in many settings, creating demand for alternative assessment modalities.

**Objective:**

To assess the feasibility and reliability of caregiver-administered anthropometric assessments of children under age 5.

**Design:**

We compared standard and caregiver-administered assessments within an ongoing nutrition trial in Zambia (NCT05120427). We developed a “no-contact” protocol whereby trained staff verbally instruct caregivers from an appropriate distance to measure the height, weight and MUAC of their children. We captured measurements of height, weight and MUAC among a sample of caregivers and infants in Zambia using both the “no-contact” protocol and a standard assessor-led protocol. We analyzed each anthropometric variable, comparing means between protocol group, the proportions yielding standardized z-scores outside the plausible ± 6SD range and the proportions of children classified stunted, underweight and wasted.

**Results:**

Anthropometric measurements were captured for 76 children using both the no-contact protocol and the standard protocol. An additional 1430 children were assessed by the standard protocol only and an additional 748 children by the no-contact protocol only. For the 76 children measured by both methods, we find no differences in average height, weight and MUAC between caregivers and interviewer assessments. The estimated kappa for the binary stunting and underweight classifications were 0.84 and 0.93, respectively. In the larger samples measured only following one protocol, we find no differences in average outcomes after adjusting for child, caregiver and household characteristics.

**Conclusions:**

Anthropometric measurement protocols administered by caregivers with verbal instruction from trained assessors are a promising alternative to standard protocols in situations where study staff are unable to come in close contact with study participants.

*Clinical trials registration* This study was conducted within a larger trial registered at clinicaltrials.gov as trial NCT05120427. https://clinicaltrials.gov/ct2/show/NCT05120427.

**Supplementary Information:**

The online version contains supplementary material available at 10.1186/s12963-024-00322-4.

## Introduction

Anthropometry is the primary means of assessing children’s nutritional status globally. In clinical settings, weight-for-age, height-for-age and mid-upper arm circumference (MUAC) are often used to diagnose chronic as well as acute malnutrition [1], and governments rely on aggregated forms of these measures to understand the population-level burden of malnutrition and to track progress toward the Sustainable Development Goals [2].

During the COVID-19 pandemic, almost all governments globally imposed social distancing requirements to restrict the spread of the SARS-CoV-2 virus. In many settings, this made it infeasible to implement standard anthropometric protocols requiring assessors to make physical contact with children. These policies also affected our then ongoing child growth trial in Zambia that required anthropometric assessments of children both at baseline and endline. The Zambian government started implementing COVID-19 response policies in March 2020 [3]. In April 2021, social distancing requirements were temporarily relieved and then re-introduced after a new COVID-19 wave in June 2021.

In order to monitor children’s nutritional status in settings requiring strict social distancing, we developed a “no-contact” anthropometric protocol whereby trained staff verbally instruct caregivers from an appropriate distance to measure the height, weight and mid-upper arm circumference (MUAC) of their children. Parental measurements of MUAC have long been used for the early detection and treatment of severe acute malnutrition. Current evidence suggests that after brief in-person trainings and provision of color-coded MUAC tapes, family members in Niger performed as well as or better than community health workers in measuring MUAC [4, 5]. Similarly positive results were reported for MUAC in Indonesia during the COVID-19 pandemic [6].

Evidence on caregiver measurement of children’s height and weight is limited. Evidence from Belgium suggests that instruction leaflets can facilitate accurate parental measurement of children’s weight and height at home [7]. A study conducted in Israel during the COVID-19 pandemic found that, with instructions, caregivers were able to measure height at home accurately except in obese and overweight children, but weight measurements were less accurate than those in the clinic [8]. A study in Australia found that caregivers were reasonably accurate in reporting child height and weight among children 4 to 11 years of age [9]. Another study conducted in the USA during the COVID pandemic found that caregiver measurements of child (2nd—4th graders) height and weight were feasible and accurate when caregivers were provided with written instructions and aided by a study team member over video calls while measuring children [10]. A recent review of under-5 remote assessments concludes that further validation studies are needed to support the larger scale use of such measurements [11].

To assess the relative accuracy of caregiver anthropometric assessments, we implemented both caregiver and interviewer assessments of children’s weight, height/length and mid-upper arm circumference in a subsample of children and compared average measurements across these groups to test for systematic measurement differences.

## Methods

### Settings and study design

This is a validation study using data collected within  a randomized controlled child growth trial in Zambia registered at clinicaltrials.gov as NCT0512047.

The study was conducted in Choma, Lusaka and Mansa districts in Zambia between April 26, 2021 and July 15, 2021. In April 2021, the government of Zambia had temporarily lifted its social distancing requirements. After a new COVID-19 wave, social distancing measures were re-introduced on June 19, 2021.

### Participants and ethical approval

All primary caregivers of children between 6 and 12 months of age residing in 281 randomly selected enumeration areas in Choma, Lusaka and Mansa districts were invited to participate in this study. Lusaka is the capital of Zambia and the largest urban agglomeration of the country. Mansa district is located in Luapula province, and among the poorest and most rural districts of the country. Choma is located in Southern Province, and comprises a mix or rural and urban communities. Given the random selection of enumeration areas for the study, the study population is fully representative of caregivers of young children in these three districts.

Written consent was obtained from all caregivers prior to the interview and assessment. The study protocol was reviewed and approved by the Ethics Committee Northwest Switzerland (AO2021-00016) as well as the University of Zambia Biomedical Research Ethics Committee (1411–2020).

### Data, variables and measurements

The primary measures of interest were children’s height, weight and MUAC. As described in the Introduction, we collected anthropometric data in two ways:

- *Standard protocol*: All children measured using standard protocols were assessed by trained study staff, using Seca scale model 784 and Seca stadiometer model 217. Given that all children were under age 2, length rather than height was measured for all children. Interviewers were trained to conduct these assessments by nutritionists from the Ministry of Health.
- *No-contact protocol:* In the no-contact protocol, the same equipment was used, but assessors were instructed to always keep at least 2 m distance from the caregiver and child. Assessors carefully instructed caregivers on how to do the measurements and provided verbal support and feedback as needed. When two adults or parents were present, they were both invited to assist with the measurements. A detailed protocol and training materials are available in the Additional file 1.

#### Protocol assignment, randomization and sample size

Standard protocols were followed from week 17 to week 22 of calendar year 2021. With the new surge of COVID cases in early 2021, it became clear that standard protocols would likely not be feasible for long in Zambia. We thus introduced the caregiver protocol gradually in calendar weeks 23–25. During this period, we randomly selected 15% of caregivers for double assessments using a simple random number draw on the tablets used by interviewers. In the randomly selected subsample that received both protocols, caregivers always measured first and interviewers subsequently did their own measurements. A total of 90 children were selected for (double-) assessments by both interviewers and caregivers.

Starting from week 26, only caregiver assessments were allowed following a decision by the Zambia Health Research Authority. No formal sample size calculation was made for the study. Ex-post, our core sample of 76 children allowed us to identify a mean difference of 2 cm in length with power 0.9. Our larger sample allowed us to identify mean differences of 0.5 cm with power 0.9.

### Bias

In our subsample where children were measured by caregivers and interviewers, measurement bias is possible if the second assessor observes, memorizes or copies the measurement of the caregiver. To avoid this, interviewers were instructed to conduct their measurements independently, and to ignore any input provided by caregivers. We also analyzed the proportion of observations with identical measurements to get a rough estimate of the potential bias through copying measurements.

### Statistical methods

We first computed descriptive statistics for the three subsamples, and tested whether the samples just assessed by caregivers differed from the sample just assessed by interviewers using standard two-sample equal means tests. Scatter plots and kernel density plots were used to compare the height, weight and MUAC measurements graphically. Ordinary least squares (OLS) regressions were used to test for mean differences in weight, height and MUAC. We estimated two sets of comparisons: a comparison of reported anthropometric measurements using our core sample of 76 children with both measures available, and a comparison of assessments done with interviewers and caregivers only (larger sample). In our analysis of the core sample, interviewer and caregiver measurements were pooled, and mean differences assessed by regressing average outcome values on an indicator of the assessment being done by the caregiver. We also estimated alignment in the binary classifications of stunting (HAZ < −2), underweight (WAZ < −2) and wasting (WHZ < −2) using the kappa statistic. We also compared the proportion of measurements outside of the plausible ± 6SD range as well as the proportion of children with missing/refused anthropometric measurements across measurement modality groups.

In our larger sample comparisons of measures taken by interviewers or caregivers only, we adjusted for caregiver age, caregiver education, caregiver height, child age, household size, wealth quintile and access to water and sanitation. Caregiver height was used as a proxy for maternal height in our analysis—98% of caregivers in our sample reported to be the biological mother of the child measured. To control for household’s socioeconomic status, we used the first principal component of the following nine binary asset indicators to divide households into five asset-based socioeconomic status quintiles: household has a concrete floor; household has a metal roof; household has piped water; household has a flush toilet, household has soap available; household has electricity; household has a TV; household has a mobile phone; household owns a car.

We also scored all measurements using the WHO’s anthropometry package, and compared the proportion of measurements outside of the plausible ± 6SD range, as well as the proportion of children stunted, underweight and wasted. Last, we analyzed measurement differences by caregiver education and children’s age.

All analysis was done using the Stata SE 16.1 software package.

## Results

**Fig. 1:**
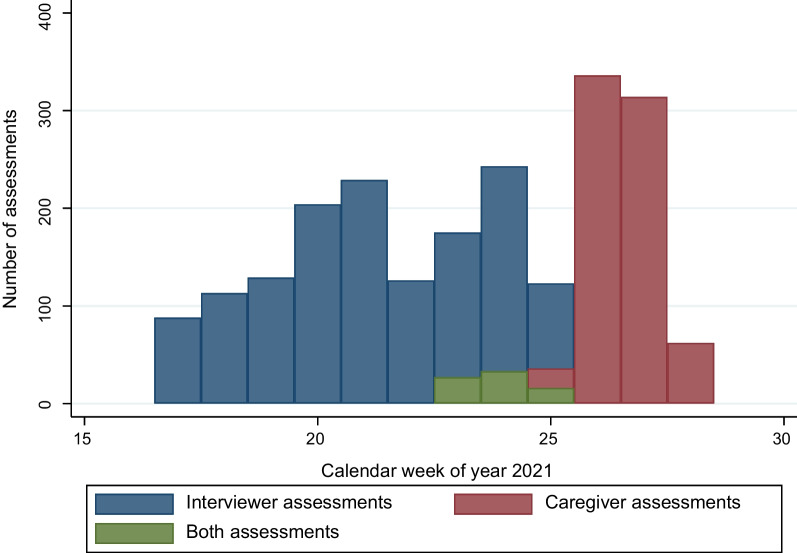
Number of caregiver and interviewer assessments per week. Figure 1 shows the number of assessments of each type by calendar week

Figure 1 summarizes the number of assessments done by calendar week and assessment modalities. A total of 2254 assessments were made between April 26, 2021, and July 15, 2021. One thousand four hundred thirty children were assessed by interviewers only, 90 were chosen to be assessed using both interviewers and caregivers and 748 children were assessed by the caregiver only. Out of the 90 chosen for double-assessment, 14 caregivers declined the invitation to measure the child in the double-assessment group; for these 14 children, only interviewer measurements were done. Additional file 1: Figure AF1 shows a flow chart for the study.

Table 1 shows average sample characteristics by group. Fifty percent of children were female and average birthweight was close to 3000*g*. On average, caregivers were 26 years old at the time of the assessment, and 75 percent were married. Average household size was 6.3. Given the design of the study and the timing of government restrictions, children assessed by caregivers only were on average 5.3 weeks older than children assessed with the standard protocol only. Caregivers in the caregiver-only group were more likely to have completed upper primary education than those in the standard protocol group.

**Table 1:** Comparison of sample characteristics

	Core sample (N = 76)		Interviewer only (N = 1430)		Caregiver only (N = 748)		Equal means test *p*-value
	Mean	SD	Mean	SD	Mean	SD	
Caregiver age	25.947	5.944	25.582	15.000	26.926	7.383	0.264
Caregiver height	155.44	5.54	156.47	6.45	157.47	6.43	**0.021**
Caregiver no education	0.013	0.115	0.009	0.095	0.009	0.096	0.950
Caregiver lower primary	0.145	0.354	0.501	0.500	0.116	0.321	**0.000**
Caregiver upper primary	0.276	0.450	0.188	0.391	0.316	0.465	**0.000**
Caregiver junior secondary	0.224	0.419	0.169	0.374	0.303	0.460	**0.000**
Caregiver senior secondary	0.263	0.443	0.107	0.309	0.201	0.401	**0.000**
Caregiver higher	0.079	0.271	0.026	0.159	0.055	0.228	**0.030**
Caregiver married	0.803	0.401	0.754	0.431	0.746	0.436	0.707
Boys under age 5	0.776	0.723	0.833	0.788	0.865	0.746	0.407
Girls under age 5	0.855	0.761	0.803	0.735	0.850	0.747	0.177
Household size	6.421	3.534	6.206	2.711	6.584	3.011	**0.014**
Household has piped water	0.395	0.492	0.506	0.500	0.293	0.455	**0.000**
Household has flush toilet	0.092	0.291	0.120	0.325	0.102	0.302	0.449
Asset quintile	2.880	1.404	3.096	1.360	2.641	1.364	0.002
Child is female	0.500	0.503	0.508	0.500	0.501	0.500	0.773
Child age in month	6.042	1.897	5.161	1.819	6.308	2.123	**0.000**
Child birthweight	2942.162	592.821	2977.302	520.899	3022.042	523.448	0.091
Child is a twin	0.079	0.271	0.043	0.202	0.029	0.169	0.201
Month of assessment	6.000	0.000	5.313	0.605	6.556	0.497	**0.000**

Table shows means and standard deviations of key variables for the cohort sample (columns 1 and 2), as well as the sample of children measured only by interviewers (columns 3 and 4) and the sample of children measured only by caregivers (columns 5 and 6). The last column shows the p-value for an equal means test between the “interviewer only” and “caregiver only” samples. *P*-values < 0.05 shown in bold font

**Fig. 2:**
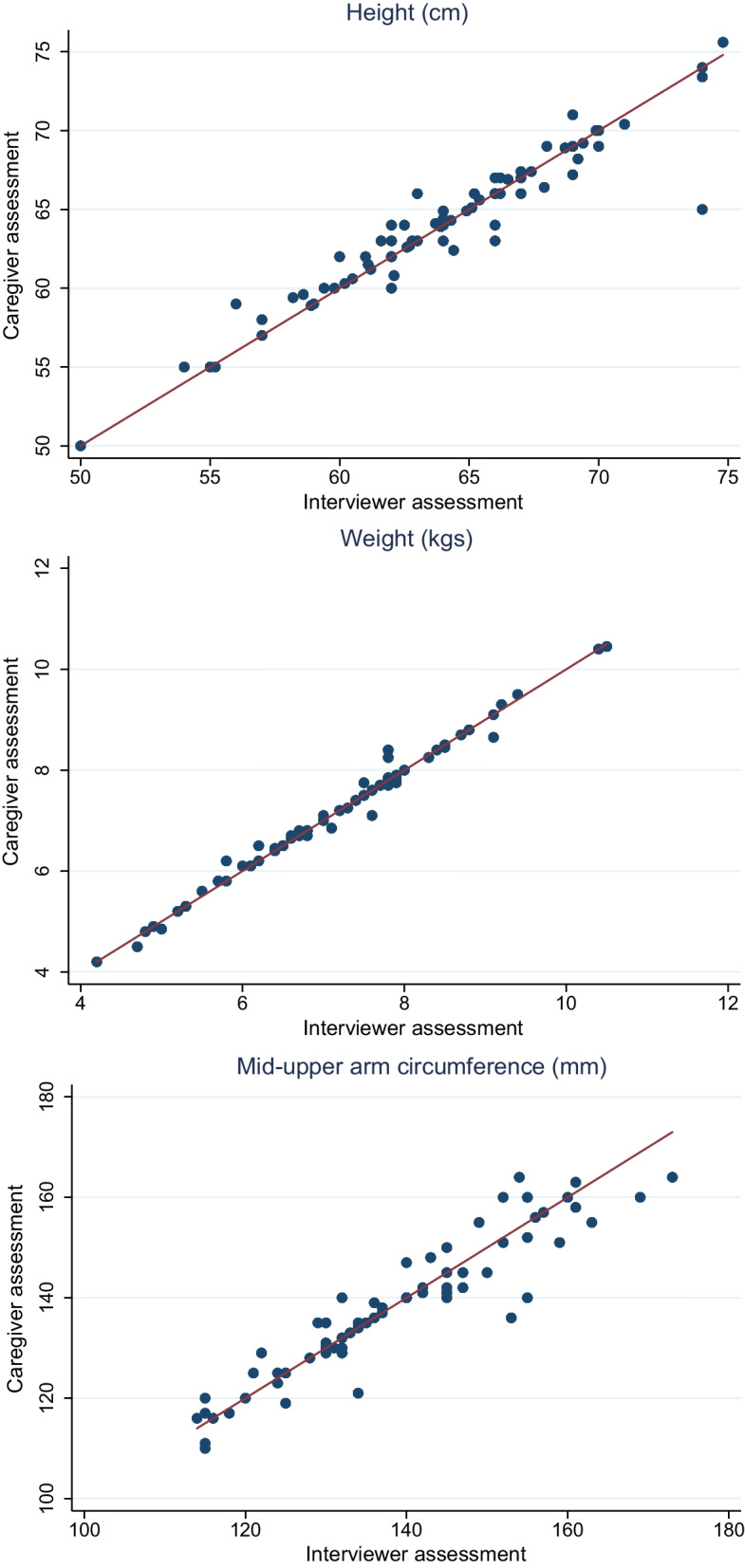
Alignment between caregiver and interviewer measurements. Figure 2 shows scatter plots comparing caregiver with interviewer assessments. Dots on the 45% degree line imply exact matches. Dots above the 45-degree line imply caregiver assessments that are higher than interviewer assessments; dots below the 45-degree lines imply caregiver measurements below interviewer measurements

Figure 2 shows the measurements of height, weight and MUAC in the core sample of 76 children done by caregivers (y-axis) relative to those completed by interviewers (x-axis). All three variables looked very similar in terms of their empirical distribution. 37% of MUAC, 34% of height and 60% of weight observations were exact matches. The correlations between caregiver and interviewer assessments were 0.95, 0.99 and 0.94 for height, weight and MUAC, respectively. The overall alignment was strongest for weight, and weakest for MUAC.

Additional file 1: Figure AF2 summarizes the overall distribution of measurements by assessment modality—the overall distribution of all three measures looked almost identical across samples.

Table 2 shows the main comparative regression results. Our null of identical average measurements (equal means) could not be rejected for any of the three measurements, with estimated mean differences of 0.5 mm for MUAC, 0.03 cm for height and 0.01 kgs for weight. Twenty-three percent of children were classified as stunted, 10.5% as underweight and only 1% of children were classified as wasted according to the interviewer measurements. The estimated kappa for the binary stunting and underweight were classifications were 0.84 and 0.93, respectively. Given that no child was rated as wasted based on caregiver measurements, kappa could not be computed for wasting. No differences in the estimated prevalences of these three measures were found between caregiver and interviewer assessments. None of the caregiver or interviewer measurements in the core sample resulted in z-scores outside of the plausible range.

**Table 2:** Regression results core sample

	Mid-upper arm circumference	Height (cm)	Weight (kgs)	HAZ < −2	WAZ < −2	WHZ < −2
Mean difference caregiver assessment	− 0.553	0.0250	0.0112	− 0.0263	− 0.0132	− 0.0132
	(0.561)	(0.161)	(0.0165)	(0.0261)	(0.0133)	(0.0131)
Mean value interviewer assessment	138.2	64.1	7.2	22.4%	10.5%	1.3%
Observations	152	152	152	152	152	152

Table shows estimated mean differences between interviewer and caregiver assessments for the six separate anthropometric outcomes. Standard errors are shown in parentheses. All models are estimated using ordinary least squares regression models with heteroscedasticity-robust standard errors to account for the non-normality of residuals clustered at the enumeration area level

Table 3 shows results from the larger comparison between interviewer-only and caregiver-only measurements. While we find higher height, weight and MUAC in the unadjusted models (which were expected due the older age of children and slightly higher socioeconomic status in the caregiver-only samples), no differences were found in average outcomes once baseline variables were adjusted for. For the binary indicators, differences were neither found for the unadjusted (age-standardized) nor the adjusted models.

**Table 3:** Measurement differences across interviewer-only and caregiver-only samples

	Outcome					
	Mid-upper arm circumference (mm)	Height (cm)	Weight (kgs)	HAZ <− 2	WAZ < 2	WHZ < 2
**Unadjusted**						
Mean difference caregiver assessment	3.916***	1.640***	0.390***	0.0212	− 0.00738	− 0.00109
(Standard error)	(0.753)	(0.248)	(0.0560)	(0.0215)	(0.0134)	(0.00892)
Sample size	2,178	2,178	2,178	2,178	2,178	2,178
**Adjusted**						
Mean difference caregiver assessment	1.374	− 0.0129	− 0.0589	0.0166	0.00311	0.0229
(Standard error)	(1.069)	(0.210)	(0.0599)	(0.0269)	(0.0182)	(0.0143)
Sample size	1,962	1,962	1,962	1,962	1,962	1,962
Mean value interviewer assessment	136.9	62.99	6.817	0.201	0.091	0.039

Table shows estimated mean differences between interviewer and caregiver assessments for the six separate anthropometric outcomes among children assessed by either standard or caregiver protocols. Standard errors are shown in parentheses. All models are estimated using ordinary least squares regression models with heteroscedasticity-robust standard errors to account for the non-normality of residuals clustered at the enumeration area level

In the larger sample, less than 1% of all observations for height and weight were outside of the plausible range; the proportion of invalid measurements was marginally smaller for caregiver assessments (0 cases for height compared to 3 cases for height among interviewers), but these differences were not statistically significant. On the other hand, the proportion of children with refused or missing data was marginally higher in the caregiver assessment group (3.1% vs. 1.4%).

Table 4 shows stratified results for height and stunting. We found no differences in measurement accuracy between caregivers with secondary or higher education versus less educated caregivers; we also found no differences by child age (less than six months vs. older).

**Table 4:** Measurement differences by caregiver education and child age groups

Sample	Height (cm)				Stunting (HAZ < − 2)			
	Primary education or less	Secondary education or higher	Age < 6 months	Age > = 6 months	Primary education or less	Secondary education or higher	Age < 6 months	Age > = 6 months
Mean difference caregiver assessment	− 0.0509	− 0.0586	− 0.114	0.0550	− 0.00647	0.0452	0.0338	0.000315
(Standard error)	(0.311)	(0.274)	(0.286)	(0.320)	(0.0395)	(0.0374)	(0.0378)	(0.0432)
Sample size	1,184	778	1,208	754	1,184	778	1,208	754

Table shows estimated mean differences between interviewer and caregiver assessments for height (columns 1–4) and a binary stunting (HAZ < − 2) indicator (columns 5–8) among children assessed by either standard or caregiver protocols. Standard errors are shown in parentheses. All models are estimated using ordinary least squares regression models with heteroscedasticity-robust standard errors to account for the non-normality of residuals clustered at the enumeration area level

## Discussion

This paper summarizes the results from a recent attempt to replace interviewer with caregiver anthropometric assessments in low-resource settings. Our main hypothesis was that caregiver measurements would vary systematically from measurements made by trained assessors. We do not find any evidence for this. Instead, the results presented here suggest that instructing caregivers to measure the height, weight and MUAC of their children in an LMIC field setting can achieve measurements that are very similar in quality to those obtained using standard protocols. In the core sample where both methods were used, the measurements were nearly identical, with highest alignment for weight (where measurements just need to be read off a scale), and lowest alignment for MUAC.

The purpose of the no-contact anthropometry method is to take measurements that approach the precision of the standard protocol while minimizing contact with study participants. This seems to have been the case in our study. The protocol developed for this study was not meant as a long-term replacement for interviewer assessment, nor designed to reduce project costs as the caregiver assessment still requires trained assessors to be present, instructing and observing caregivers as they measure their children. In this sense, the protocol used in this study is quite different from “fully remote” assessments, where parents do measurements by themselves following instructions provided remotely via phone, mail or other channels. In terms of implementation cost, the caregiver assessments will increase survey length and may thus increase survey costs at the margin. The main advantage of this method—which was particularly important during COVID-19—is that it allows measurements without direct physical contact to children or caregivers. From an implementation perspective, it is also possible that caregiver assessment is more pleasant for children, who likely prefer measurement by their parents to measurements by unfamiliar study staff. We did not collect data on children’s reaction to the measurements as part of this study.

However, we did collect general feedback from interviewers who performed the assessments with caregivers. Their feedback was overwhelmingly positive and they uniformly expressed their confidence in this approach being feasible at larger scale as well as for older children. While there are several new technologies that may make no-contact feasible in the future without caregiver involvement such as three-dimensional scanning [12], these technologies are not ready for large scale use yet. In the meantime, caregiver-administered measurements might be a viable and attractive way of collecting anthropometric data both in clinical and research settings, even in non-pandemic situations.

The study presented here has a few limitations. First, we only were able to collect two measurements for 76 children, which limited our statistical power to detect measurement differences. While the larger two-sample comparison is more vulnerable to potential confounding bias (despite the large number of covariates included), having the two larger samples did allow us to test and reject for systematic differences in measurements and classifications across measurement modalities. In terms of household and caregiver characteristics, the 76 children selected seem to be comparable to the larger study population. As mentioned above, all children assessed were also relatively young. In practice, measuring infants tends to be more difficult than measuring older kids (especially for length/height), which suggests that differences could be even smaller for older children. It is also not clear if caregivers would be equally willing to engage with such measurements in other settings—even though Zambia seems fairly representative for a larger group of lower middle-income countries. Further validation studies will be needed to prove the external validity of the results presented here. In our study, fourteen of the 90 caregivers (15.5%) in the initial sample declined the invitation to measure their children. The refusal rate was substantially reduced to 3.1% in the caregiver-only sample. One explanation for this difference is that in the context of a larger RCT, the interviewers administering double measurement knew they would get at least one of the two measurements and thus satisfy the main objective for the RCT. They were thus likely more willing to accept a reason to not do the caregiver measurement. In the caregiver-only sample, interviewers did not have the safety net of the second measurement and may have been more persistent in finding solutions to obstacles. A further limitation of the study is that the interviewers instructing caregivers also did the second assessments, which may have introduced some bias toward aligned measurements. Given that we find exactly matching heights only in about one third of cases, copying of results does not appear to have been a common pattern—having a more neutral second assessor would nevertheless be preferable for future studies.

## Conclusions

The results of this study suggest that caregiver-led anthropometric assessments of children under age 5 are feasible and seem equally reliable as measurements made by trained assessors. We found that caregiver assessments did not require much additional time or other resources and should thus be considered as an effective way to reduce infectious disease exposure for both study staff and families participating in research.

### Supplementary Information

**Additional file 1.** Supplementary Materials. **Figure AF1:** Flow Chart. **Figure AF2:** Overall distribution of height, weight and MUAC in core sample by assessment type (N = 76).

## Data Availability

Described in the manuscript, code book and analytic code will be made publicly and freely available upon request to the first author at guenther.fink@swisstph.ch.

## Abbreviations

MUAC: Mid-upper arm circumference
HAZ: Height-for-age z-score
WAZ: Weight-for-age z-score
