# Supplementary material for: Feasibility of caregiver-administered anthropometric measurements of children under age 5: evidence from Zambia
Source: Popul Health Metr. 2024 Jan 31;22:2. doi: 10.1186/s12963-024-00322-4 (PMC10829329; doi:10.1186/s12963-024-00322-4)
Supplement: Supplementary file 1 — Additional file 1. Supplementary Materials. Figure AF1: Flow Chart. Figure AF2: Overall distribution of height, weight and MUAC in core sample by assessment type (N = 76). [file 12963_2024_322_MOESM1_ESM.docx]

**Supplementary Materials Figure AF1: Flow Chart**

**
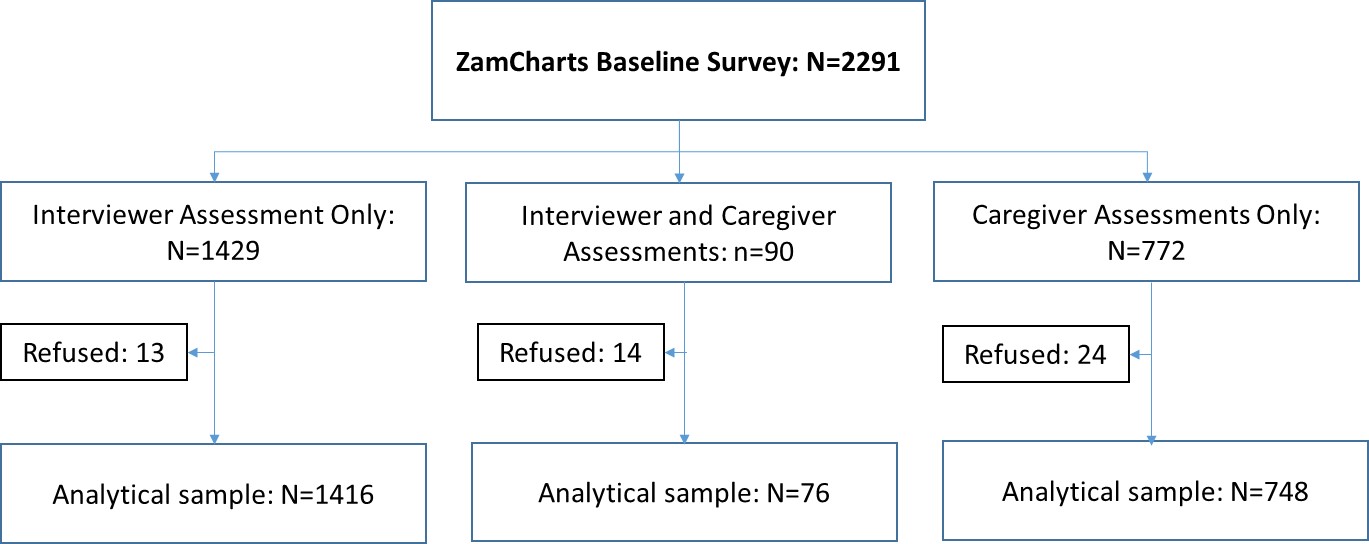
**

*Supplementary Materials Figure AF1 footer:* Figure shows allocation of the full ZamCharts study sample into assessment modalities as well as number of refusals in each arm.

**Supplementary Materials Figure AF2: Overall distribution of height, weight and MUAC in core sample by assessment type (N=76)**


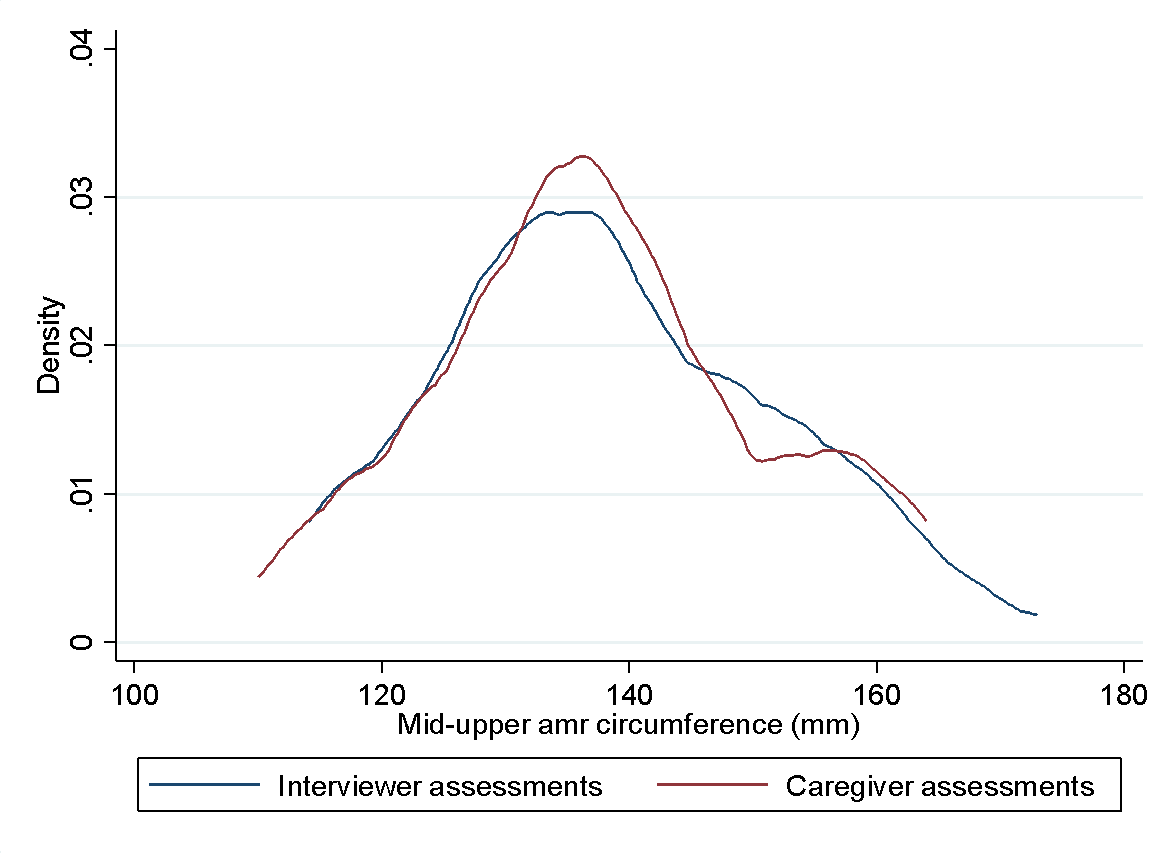


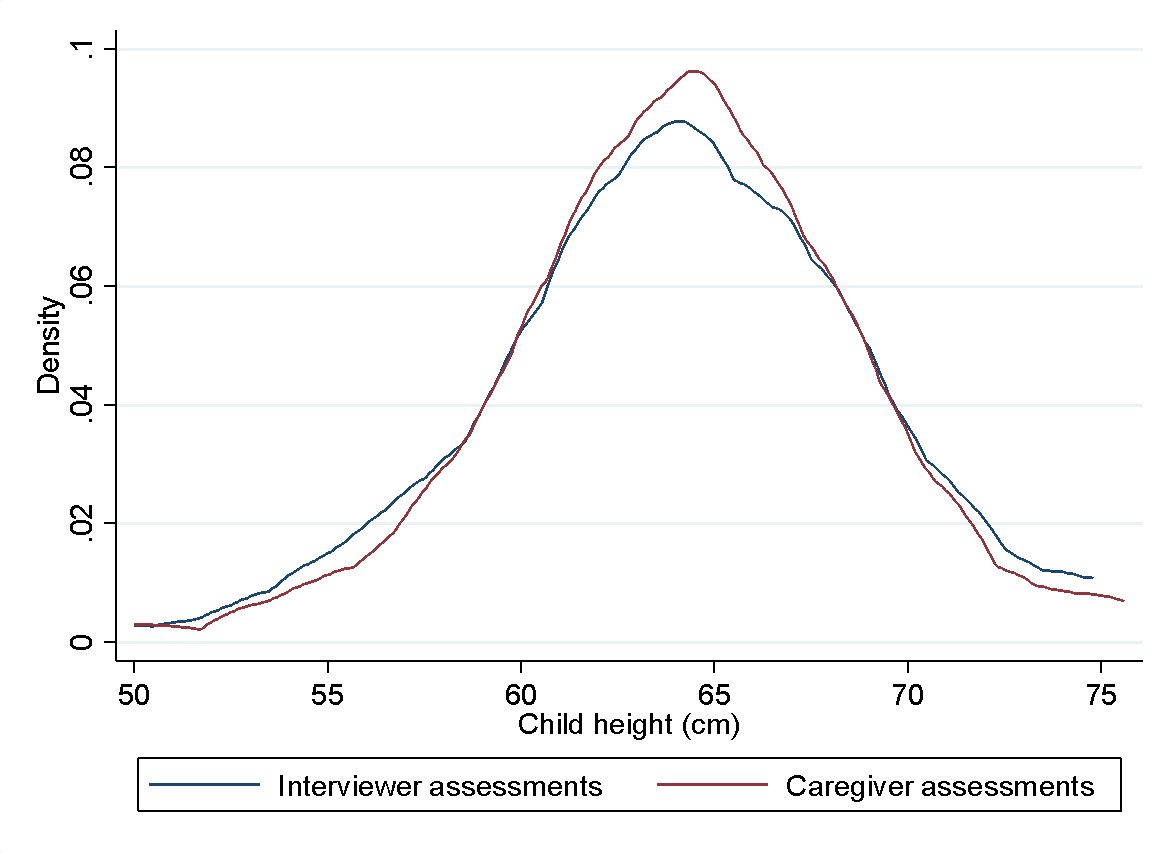


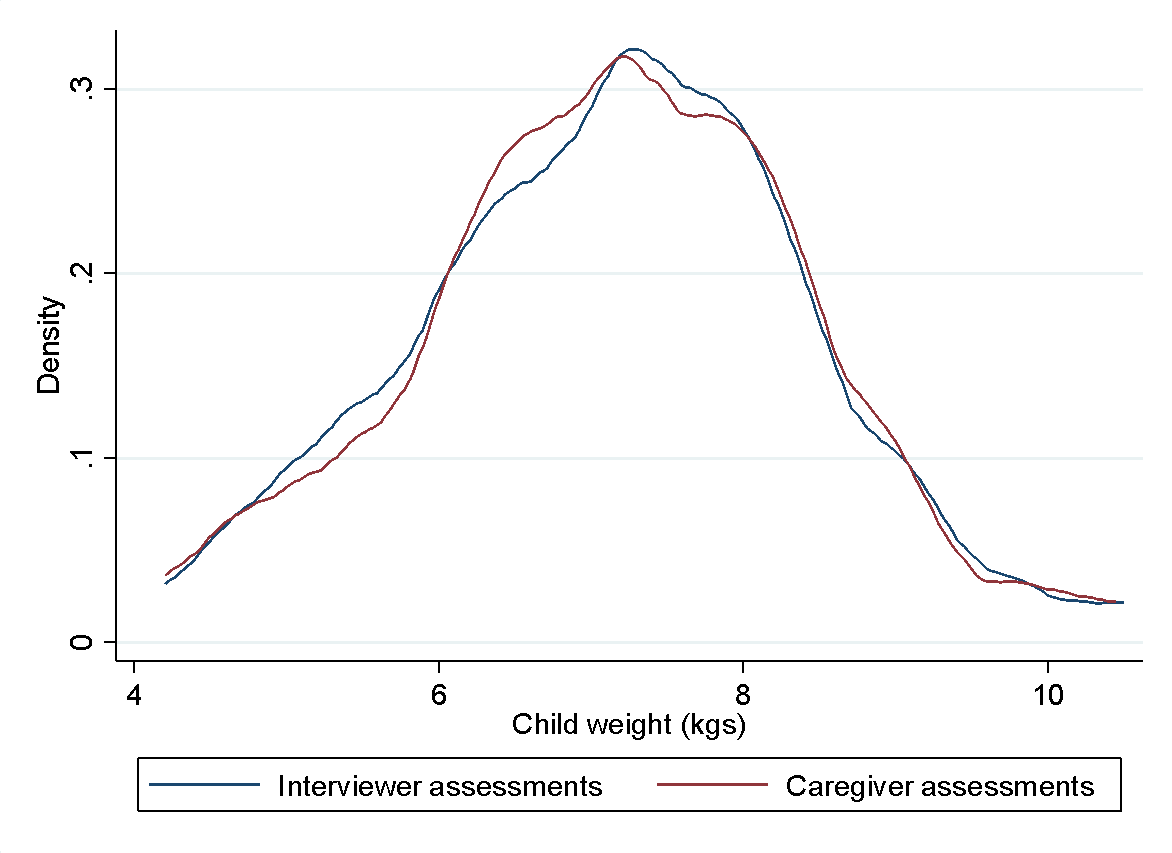


*Supplementary Materials Figure AF2 footer:* Figure shows kernel density plots for MUAC, height and weight. Based on 76 caregiver as well as 76 interviewer assessments.
